# *Rhabdophis tigrinus* (Yamakagashi) Bites in Japan Over the Last 50 Years: A Retrospective Survey

**DOI:** 10.3389/fpubh.2021.775458

**Published:** 2022-01-10

**Authors:** Toru Hifumi, Atsushi Sakai, Akihiko Yamamoto, Kazunori Morokuma, Norio Otani, Motohide Takahashi, Manabu Ato

**Affiliations:** ^1^Department of Emergency and Critical Care Medicine, St. Luke's International Hospital, Tokyo, Japan; ^2^The Japan Snake Institute, Gunma, Japan; ^3^Management Department of Biosafety and Laboratory Animal, National Institute of Infectious Disease, Tokyo, Japan; ^4^Toxin and Biologicals Research Laboratory, Kumamato Health Science University, Kumamoto, Japan; ^5^Kikuchi Quality Control Department, KM Biologics Co., Ltd., Kumamoto, Japan; ^6^Leprosy Research Center, National Institute of Infectious Diseases, Tokyo, Japan

**Keywords:** snake envenomation, antivenom, disseminated intravascular coagulation, neglected tropical disease, Yamakagashi

## Abstract

**Introduction:** *Rhabdophis* snakes, which include 27 species, are rear-fanged venomous snakes that are widely distributed from India to East Asia and Russia. Severe envenomation by *R. tigrinus* (Yamakagashi snake) in Japan and *R. subminiatus* in Southeast Asia has been reported. The epidemiology of *R. tigrinus* bites, such as geographical features, the incidence, and changes in the number of bites over time have not been comprehensively examined. Hence, we intended to clarify the epidemiological features of *R. tigrinus* bites through a careful review of scientific data over the last 50 years in Japan.

**Methods:** Patient records of *R. tigrinus* bites between 1971 and 2020 at the Japan Snake Institute were examined retrospectively. The following were ascertained: patient characteristics, clinical symptoms, laboratory data, treatment-related factors, and hospital mortality. These variables were compared in the antivenom and the without-antivenom groups.

**Results:** Over the 50-year study period, 43 *R. tigrinus* bites, including five fatal cases, were encountered. Severe cases of *R. tigrinus* bites have been treated with antivenom since 1985; however, fatalities occurred in 2006 and 2020. *R. tigrinus* bite cases have been well-distributed in the western part of Japan since 2000. The mortality rate in the antivenom group was significantly lower in the patient group that was not administered the antivenom (0 vs. 23.8%, *p* = 0.048).

**Conclusion:** This study clarified the epidemiology of *R. tigrinus* bites in Japan over a 50-year period. Almost all severe cases of *R. tigrinus* bites have been treated with the antivenom in the current situation, and fatalities occurred in cases not treated with the antivenom. It is important to diagnose *R. tigrinus* bites in the early phase of the clinical course. The antivenom, the definitive treatment for *R. tigrinus* bites, is an unapproved drug. Hence, approval needs to be obtained for the drug.

## Introduction

Snakes of genus *Rhabdophis* include 27 species of rear-fanged venomous snakes that are widely distributed from India to East Asia and Russia. Severe envenomation by *R. tigrinus* (Yamakagashi snake) in Japan and *R. subminiatus* in Southeast Asia have been reported (1–5). Their bites lead to venom-induced consumption coagulopathy (VICC), resulting in hemorrhage (6, 7). The experimental use of antivenom against *R. tigrinus* bites was first tested in 1985 on immunized rabbits (1). Currently, the antivenom against *R. tigrinus* bites is provided for use only in clinical research because it is an unapproved drug in Japan (1, 4, 8). Our research group is currently working on the supply of antivenom as a serum therapy against *R. tigrinus* bites (9–13).

The clinical characteristics of *R. tigrinus* bites in Japan have been studied as part of a national survey from 2000 to 2013 (14), and its treatment (i.e., efficacy of *R. tigrinus* antivenom) has also been evaluated from 1973 to 2013 (15). However, the epidemiology of *R. tigrinus* bites, including the change of the geographical distribution and incidence over the time has not been well-studied.

In this study, we aimed to clarify the epidemiological features of *R. tigrinus* bites in Japan by retrospectively reviewing scientific data over the last 50 years.

## Materials and Methods

This study was approved by the Institutional Review Board, St. Luke's International Hospital, with approval number 21-R093.

### Patients and Setting

In 1968, the Japan Snake Institute (JSI) was founded in the Gunma Prefecture to conduct research and provide enlightenment on snakes (15). This institute provides medical support to physicians handling patients with venomous snake bites where diagnosis is sometimes achieved based on patient history, laboratory analyses, and clinical symptoms. In this study, we conducted a retrospective review by collecting clinical data and all recorded cases of *R. tigrinus* bites at JSI from January 1, 1971, to December 31, 2020.

### Diagnosis of *R. tigrinus* Bites

The diagnosis of *R. tigrinus* bites was based on the patients' description of the snakes and the presence of VICC in patients with hypofibrinogenaemia.

### Data Collection

Data on age, sex, date of injury (year), place of injury, clinical symptoms, laboratory analyses, treatment-related factors, and hospital mortality were recorded.

### Primary Data Analyses

#### Statistical Analysis

To compare the data pertaining to patient characteristics, treatment-related factors and outcomes between the antivenom patient group and the without-antivenom group, the Mann–Whitney *U*-test or the Chi square (χ^2^) test was used. Furthermore, the Fisher exact test for 2 × 2 categorical variables was used when appropriate. *P*-values < 0.05 were considered statistically significant. All analyses were completed using the JMP version 11 (SAS, Cary, North Carolina) software.

## Results

### Demographic and Clinical Characteristics of the Patients

Table 1 summarizes the patient characteristics. Over the 50-year study period, 43 *R. tigrinus* bite patients were recognized. The median age was 37 years, and 97.7% were men.

**Table 1:** Population characteristics of the patients.

**Characteristics**	**All (*n* = 43)**
Age (years)	37 (12–57)
Sex, male	42 (97.7)
**Clinical symptoms**	
Nasal bleeding	4 (10.8)
Gum bleeding	15 (40.5)
Bleeding from the bite sites	29 (80.6)
Headache	6 (16.7)
**Laboratory data**	
Platelet counts (× 10^4^/mm^3^)	10.9 (5.7–15.6)
Fibrinogen (mg/dL)	35 (20–50)
PT-INR	4.4 (2.3–7)
FDP (μg/mL)	200 (80.3–320)
**Treatment**	
PE	4 (10.8)
**Outcome**	
Mortality	5 (11.9)

*Data are presented as median (interquartile, IQR) for continuous variables and n (percentage) for categorical variables*.

*PT-INR, prothrombin time international ratio; FDP, fibrinogen degradation products; PE, plasma exchange*.

*Missing data: Age = 0, Sex male = 0, Nasal bleeding = 6, Gum bleeding = 6, Bleeding from the bite sites = 7, Headache = 7, Platelet counts = 19, Fibrinogen = 19, PT-INR = 23, FDP = 16, PE = 6, Mortality = 1*.

### Geographical Features of *R. tigrinus* Bites

**Figure 1:**
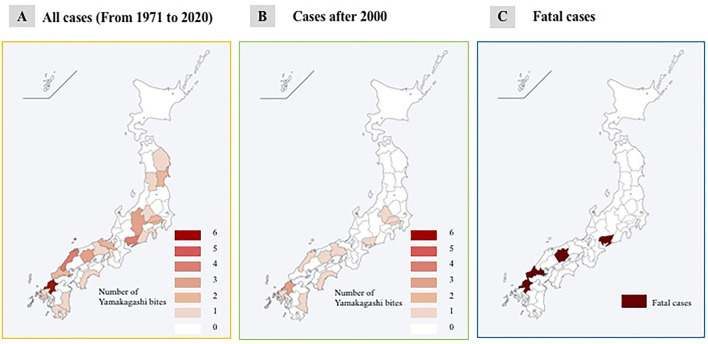
Geographical features of *R. tigrinus* bites. **(A)** All cases (from 1971 to 2020). **(B)** Cases after 2000. **(C)** Fatal cases.

*R. tigrinus* bite cases were found in almost all parts of Japan, except Hokkaido and Okinawa areas, and have been distributed in the western part of Japan since 2000. Fatalities have been limited to the western part, and specifically, there have been two fatalities in the Okayama Prefecture (Figures 1A–C).

### Trends in *R. tigrinus* Bites Over the Last 50 Years

**Figure 2:**
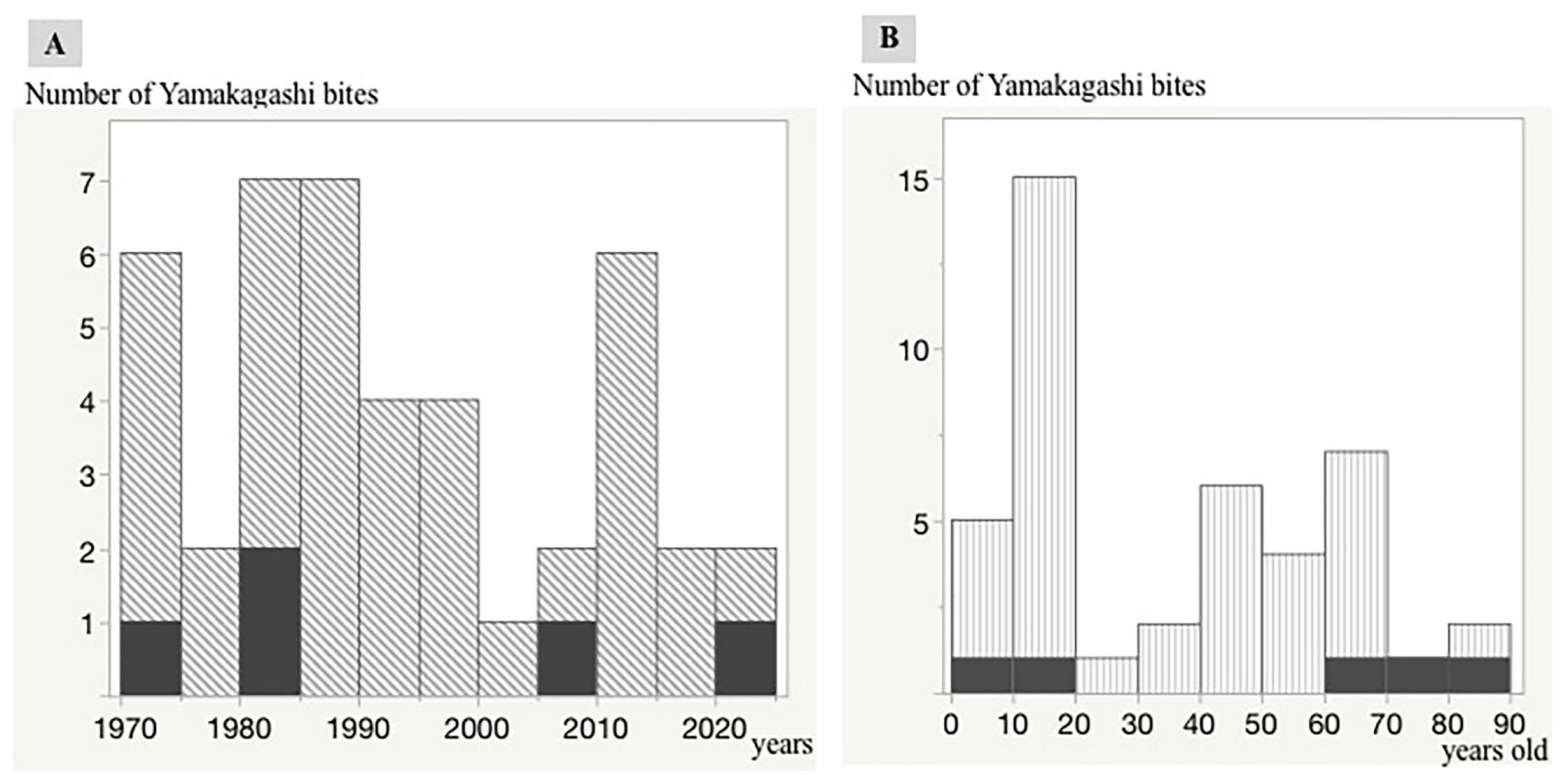
Distribution of *R. tigrinus* bites in Japan. Trends in *R. tigrinus* bites over the last 50 years. The black regions indicate fatal cases. A single vertical bar indicates a 5-year period (1970–1974, 1975–1979, 1980–1984, 1985–1999, 2000–2004, 2005–2009, 2010–2014, 2015–2019, and 2020). Relationship between age and number of *R. tigrinus* bite cases. The black regions indicate fatal cases. A single vertical bar indicates a 10-year period (0–10, 11–20, 21–30, 31–40, 41–50, 51–60, 61–70, 71–80, and 81–90). **(A,B)** Number of Yamakagashi bites.

During the past 50 years, there have been several cases with *R. tigrinus* bites at intervals of 5 years. A fatal case tends to occur approximately once every 10 years (Figure 2A).

### Relationship Between Age and Number of *R. tigrinus* Bite Cases

The relationship between age and the number of *R. tigrinus* bites is characteristic, with a clear peak in the teens. Fatal cases were observed in the elderly and children (Figure 2B).

### Trends in Patients Treated With Antivenom Over the Last 50 Years

**Figure 3:**
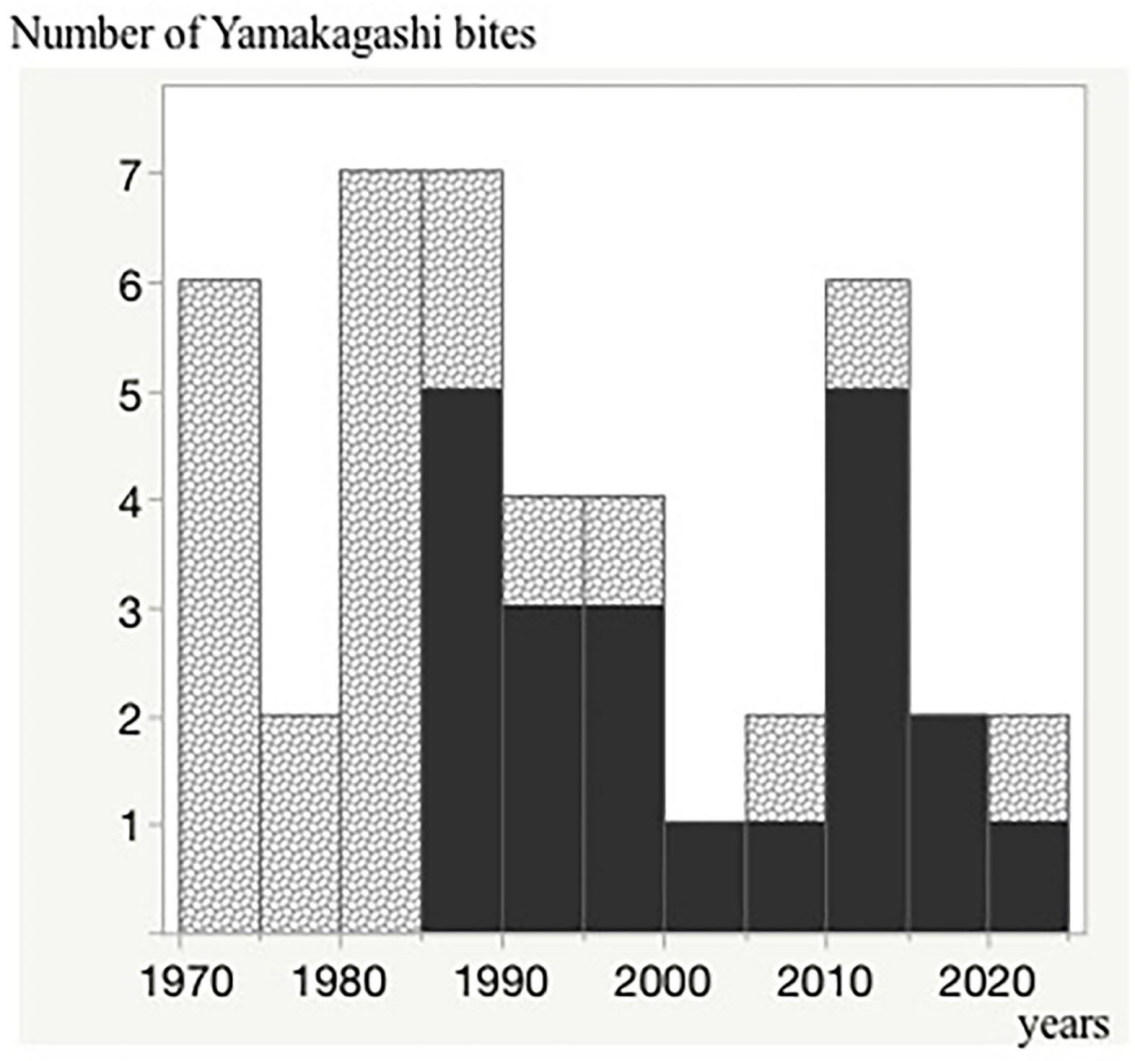
Trends in patients treated with the antivenom over the last 50 years. The black regions indicate the antivenom administrated cases. A single vertical bar indicates a 5-year period (1970–1974, 1975–1979, 1980–1984, 1985–1999, 2000–2004, 2005–2009, 2010–2014, 2015–2019, and 2020). Number of Yamakagashi bites.

The first case of antivenom administration was in 1985, and almost all severe cases have been treated with the antivenom since then (Figure 3).

### Comparison of Clinical Characteristics Between the Antivenom and Without-Antivenom Patient Groups

Table 2 presents the clinical features observed in the antivenom and without-antivenom groups. No significant differences were observed in the baseline characteristics and results of laboratory analyses between the two groups. The mortality rate in the antivenom group was significantly lower in the patient group that was not administered the antivenom (0 vs. 23.8%, *p* = 0.048).

**Table 2:** Comparison of clinical characteristics between the antivenom and without-antivenom patient groups.

**Characteristics**	**Antivenom group (*n* = 21)**	**Without-antivenom group (*n* = 22)**	***P***
Age (years)	20 (11–51)	42 (12–61)	0.451
Sex, male	20 (95.2)	22 (100)	0.488
**Clinical symptoms**			
Nasal bleeding	1 (4.8)	3 (18.8)	0.300
Gum bleeding	8 (38.1)	7 (43.8)	0.749
Bleeding from the bite sites	17 (85.0)	12 (75.0)	0.675
Headache	3 (15.0)	3 (18.8)	1.00
**Laboratory data**			
Platelet counts ( × 10^4^/mm^3^)	12.5 (7.3–17.3)	9.5 (2.9–12.2)	0.213
Fibrinogen (mg/dL)	35 (28–50)	33 (10–52)	0.532
PT-INR	5.8 (2.8–7)	2.6 (2–5.5)	0.094
FDP (μg/mL)	262 (173–397)	160 (70–284)	0.070
**Treatment**			
PE	1 (4.8)	3(18.8)	0.296
**Outcome**			
Mortality	0 (0.0)	5 (23.8)	0.048

*Data are presented as median (interquartile, IQR) for continuous variables and n (percentage) for categorical variables*.

*PT-INR, prothrombin time international ratio; FDP, fibrinogen degradation products; PE, plasma exchange*.

*Missing data (Antivenom group): Age = 0, Gender male = 0, Nasal bleeding = 0, Gum bleeding = 0, Bleeding from the bite sites = 0, Headache = 1, Platelet counts = 9, Fibrinogen = 4, PT-INR = 8, FDP = 4, PE = 0, Mortality = 0*.

*Missing data (Without-antivenom group): Age = 0, Gender male = 0, Nasal bleeding = 6, Gum bleeding = 6, Bleeding from the bite sites = 7, Headache = 6, Platelet counts = 10, Fibrinogen = 15, PT-INR = 15, FDP = 12, PE = 6, Mortality = 1*.

### Details of Fatal Cases

Details of fatalities are provided in Table 3. There have been two deaths since 2000 after the development of the antivenom. Our research group obtained the relevant clinical information after death or brain death in the two fatal cases. None of the fatal cases were treated with *R. tigrinus* antivenom. Cerebral hemorrhage was observed in least three out of five cases.

**Table 3:** Clinical characteristics of fatal cases among *R. tigrinus* bite patients in Japan (1971–2020).

**Characteristics**					**Clinical symptoms**				**Laboratory data**				**Treatment**	**Outcome**	
**Case**	**Year**	**Area**	**Age**	**Sex**	**Nasal bleeding**	**Gum bleeding**	**Bleeding from the bite sites**	**Headache**	**Platelet counts (× 10^**4**^/mm^**3**^)**	**Fibrinogen (mg/dL)**	**PT-INR**	**FDP (μg/mL)**	**Antivenom**	**Hospital stay**	**Brain bleeding**
1	2020	Okayama	80	M	0	0	1	0	11	50	2.12	N/A	0	1	1
2	2006	Fukuoka	75	M	0	0	1	0	1.8	50	2.62	271.5	0	6	1
3	1984	Aichi	14	M	0	0	1	1	9	5	N/A	40	0	10	1
4	1982	Yamaguchi	6	M	0	0	0	0	N/A	N/A	N/A	N/A	0	4	N/A
5	1972	Okayama	61	M	0	1	1	1	N/A	20	N/A	N/A	0	60	N/A

*PT-INR, prothrombin time international ratio; FDP, fibrinogen degradation products; DIC, disseminated intravascular coagulation; PE, plasma exchange; N/A, not available*.

## Discussion

Over the 50-year study period, 43 cases of *R. tigrinus* bites, including five fatalities, were identified. Patients with *R. tigrinus* bites have been treated with the antivenom since 1985, and fatalities occurred in 2006 and 2020 in patients who were not treated with the antivenom.

Our survey showed that *R. tigrinus* bite cases have been well-distributed in the western part of Japan since 2000. Fatalities have also been limited to the western part. This finding is similar to the cases of pit viper bites reported previously (9, 10) and could be attributed to the short activity period due to the long winter experienced in the Tohoku and Hokuriku regions. Although the number of *R. tigrinus* cases is uncertain, it is believed that its density is less in the Tohoku and Hokuriku regions than in the western part of Japan mainly due to the varying climatic situations. The density of frogs, which are their bait, is also less in these regions than in western Japan, which affects the snake population.

The population of *R. tigrinus* is getting smaller (1). However, the number of *R. tigrinus* bites has remained almost constant. This discrepancy may be because many children, especially those who intentionally play with snakes, are bitten repeatedly, which allows the venom to enter the body and result in toxic bites (16).

Since the development of the antivenom for *R. tigrinus* bites in 1985, the number of fatalities has been reduced owing to the aggressive administration of the antivenom. However, two fatalities occurred in elderly people, both of whom were confirmed to have died from complications related to *R. tigrinus* bites. No fatalities have been observed among patients treated with the antivenom, which suggests that it is important to diagnose *R. tigrinus* bites in patients with unexplained severe coagulopathy (17) in the early phase of the clinical course. Raising awareness not only in the departments of emergency and intensive care but also in the fields of pediatrics and neurosurgery is imperative.

This study clarified the epidemiology of *R. tigrinus* bites in Japan over a 50-year period. The antivenom was experimentally manufactured by regional health laboratories; however, its sterility and safety were not guaranteed (1). The Ministry of Health, Labor and Welfare of Japan, launched a research group to evaluate the safety and efficacy of the antivenom and to collect information on *R. tigrinus* bites in 2013 (18). The antivenom is stocked in some institutions and supplied to the hospital under the direction of the principal investigator in this research. Clinical characteristics and details of treatment, including the adverse effects of the antivenom, are recorded for all cases and analyzed to ensure proper safety (14). Therefore, based on the 50-year data, we plan to negotiate with the relevant authorities to obtain approval for the drug. Furthermore, the *R. tigrinus species* is not only found in Japan but also in Asia, and it is necessary to use these data to educate people in China and other countries where it is considered a non-toxic bite.

We observed some limitations in this study. Ours was a retrospective study, and a small sample size was utilized. There is the possibility of a selection bias since the cases reported were limited to our center only. The consistency of the type of data collected data, and the accuracy of data varied over the 50-year period, particularly in case of the laboratory data varies for the period of 50 years. Furthermore, some cases may have remained undiagnosed or misdiagnosed due to the unusual symptoms associated with this rare snakebite.

## Conclusion

Over the 50-year study period, 43 cases of *R. tigrinus* bites, including five fatal cases, were identified. Almost all severe cases of *R. tigrinus* bites have been treated with the antivenom in the current situation, and fatalities occurred in patients who were not treated with the antivenom. It is important to diagnose *R. tigrinus* bites in patients with unexplained severe coagulopathy in the early phase of the clinical course. The antivenom, the definitive treatment for *R. tigrinus* bites, is an unapproved drug. Hence, it is necessary to obtain approval for this drug.

## Data Availability Statement

The raw data supporting the conclusions of this article will be made available by the authors, without undue reservation.

## Ethics Statement

The studies involving human participants were reviewed and approved by St. Luke's International Hospital. Written informed consent to participate in this study was provided by the participants' legal guardian/next of kin.

## Author Contributions

TH and AS was responsible for conception of the article and drafted and revised the manuscript. AY, KM, NO, MT, and MA revised the manuscript. All authors read and approved the final manuscript.

## Funding

This work was supported by the Research Program on Emerging and Re-emerging Infectious Disease from the Japan Agency for Medical Research and Development (AMED) 20fk0108101.

## Conflict of Interest

KM was employed by company KM Biologics Co., Ltd. The remaining authors declare that the research was conducted in the absence of any commercial or financial relationships that could be construed as a potential conflict of interest.

## Publisher's Note

All claims expressed in this article are solely those of the authors and do not necessarily represent those of their affiliated organizations, or those of the publisher, the editors and the reviewers. Any product that may be evaluated in this article, or claim that may be made by its manufacturer, is not guaranteed or endorsed by the publisher.
